# Safety and feasibility of hyperkalemic cardioplegia with diazoxide in cardiac surgery: The Cardioplegia with Diazoxide in Cardiac Surgery Trial

**DOI:** 10.1016/j.xjon.2026.101689

**Published:** 2026-02-19

**Authors:** AlleaBelle Bradshaw, Jessica B. Briscoe, Lisa Fornaresio, Zyriah Robinson, Michelle Carvajal Villalba, Shivani Shirodkar, Hamza Aziz, Jennifer S. Lawton

**Affiliations:** Division of Cardiac Surgery, Department of Surgery, Johns Hopkins University, Baltimore, Md

**Keywords:** diazoxide, cardioprotection, K_ATP_ channel

## Abstract

**Background:**

We have demonstrated cardioprotection by the adenosine triphosphate–sensitive potassium (K_ATP_) channel opener diazoxide in multiple animal models in an effort to reduce myocardial stunning following cardiac surgery. These translational findings supported this first-in-human Phase I safety and feasibility trial evaluating diazoxide as an additive to hypothermic, hyperkalemic cardioplegia.

**Methods:**

In this Food and Drug Administration–approved safety and feasibility trial, 30 patients undergoing nonemergent cardiac surgery (coronary artery bypass, aortic, or valve) received intracoronary diazoxide in the first dose of hypothermic, hyperkalemic cardioplegia at the time of cross-clamp placement. Safety and clinical endpoints, including a novel definition of myocardial stunning (need for inotropic support for >24 and <72 hours), myocardial enzymes, change in ejection fraction, and hemodynamic parameters, were collected.

**Results:**

Thirty patients received intracoronary diazoxide. The Society of Thoracic Surgeons Predicted Risk of Mortality ranged from 0.3% to 8.0%. An increase in mean arterial pressure was noted with diazoxide administration (mean, 59.8 mm Hg before vs 62.0 mm Hg after). The mean time to arrest was 80 ± 43 seconds. Two patients (6.7%) had myocardial stunning. Mean peak lactate was 5.6 ± 5.1, peak creatine kinase was 939 ± 588 U/L, and peak troponin was 12,531 ± 18,615 ng/L. An increase in left ventricular ejection fraction of 2.1 ± 4.9% (prebypass to postbypass by transesophageal echocardiography) was noted.

**Conclusions:**

Intracoronary diazoxide is safe and feasible in patients undergoing nonemergent cardiac surgery.

## Graphical Abstract

**Figure:**
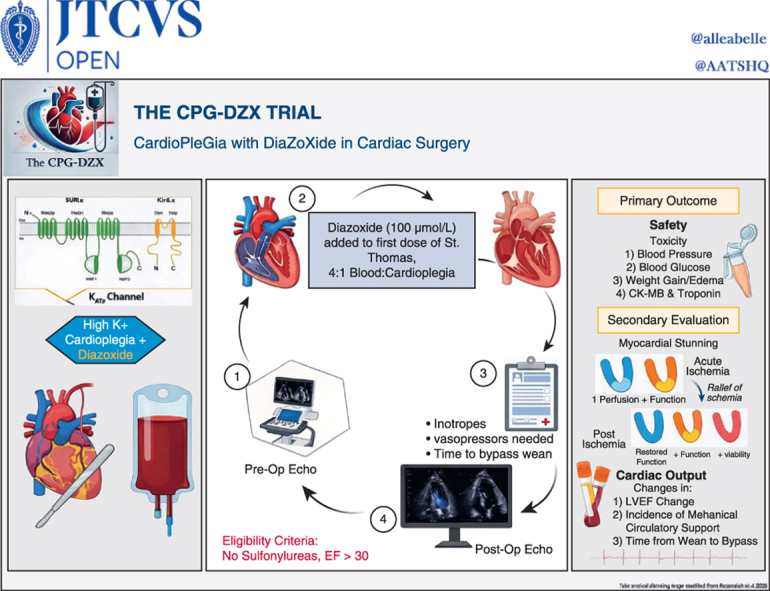

**Figure:**
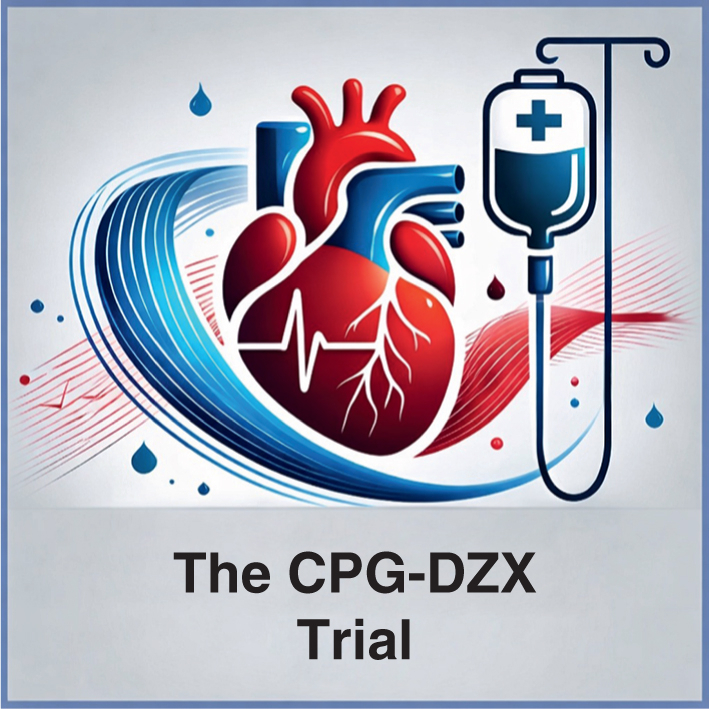
Safety and feasibility of hyperkalemic cardioplegia with diazoxide in cardiac surgery.

**Central Message**

The first human trial of the K_ATP_ channel opener diazoxide added to hypothermic hyperkalemic cardioplegia demonstrates safety and feasibility.

**Perspective**

Myocardial stunning after cardiac surgery is common and associated with adverse outcomes. K_ATP_ channel openers, such as diazoxide, mimic ischemic preconditioning and may reduce stunning. This Phase I study demonstrates safety and feasibility of diazoxide, supporting advancement to a randomized clinical trial (RCT).

Patients undergoing cardiac surgery are at risk for postoperative myocardial stunning, a postischemic mechanical dysfunction that persists despite restoration of blood flow.^1^^,^^2^ The majority of patients who undergo cardiac surgery experience stunning, which requires increased pharmacologic and mechanical support and increases health care costs.^3^^,^^4^ Stunning also is associated with poor outcomes, including increased mortality.^5^ Despite the use of cardioplegia and hypothermia, myocardial injury still occurs.^6^ We have shown that 3 insults contribute to this injury: hyperkalemic cardioplegia, global ischemia during cross-clamp application, and the presence of an already compromised myocardium.^7^, ^8^, ^9^, ^10^

A change in the status quo of myocardial protection is needed, given the growing demand for improved strategies to protect the heart from ischemia-reperfusion injury.^11^^,^^12^ An increasing percentage of patients are older and have multiple comorbid conditions, present while in shock, have undergone prior percutaneous coronary intervention, and have reduced left ventricular ejection fraction (LVEF).^13^ These patients have more complex disease that is associated with longer global ischemic time and higher risk of myocardial stunning. Thus, there is a tremendous unmet need for improved myocardial protection strategies.^14^

The cardioprotective effect of the adenosine triphosphate-sensitive potassium (K_ATP_) channel opener diazoxide has been demonstrated in models ranging from isolated mitochondria to large animals.^15^ Diazoxide’s effect mimics ischemic preconditioning.^16^, ^17^, ^18^, ^19^ By giving the drug before the ischemic insult of cardiac surgery, the myocardium appears to be “conditioned” and thus sustains less injury from global ischemia.^20^^,^^21^ The exact subcellular mechanism remains to be elucidated, but it is believed to involve a putative mitochondrial K_ATP_ channel and succinate dehydrogenase inhibition.^22^, ^23^, ^24^

With robust translational data supporting myocardial protection with diazoxide^25^, ^26^, ^27^ the US Food and Drug Administration (FDA) approved the use of intracoronary diazoxide (IND 156146) in this Phase I study in humans. The primary outcome was to evaluate the safety and feasibility of using intracoronary diazoxide as an additive to hypothermic hyperkalemic cardioplegia in adult patients undergoing elective cardiac surgery. We hypothesized that this use of diazoxide is safe and feasible. The secondary outcomes of interest were collected to establish endpoints for a future Phase III RCT (Figure 1).

**Figure 1:**
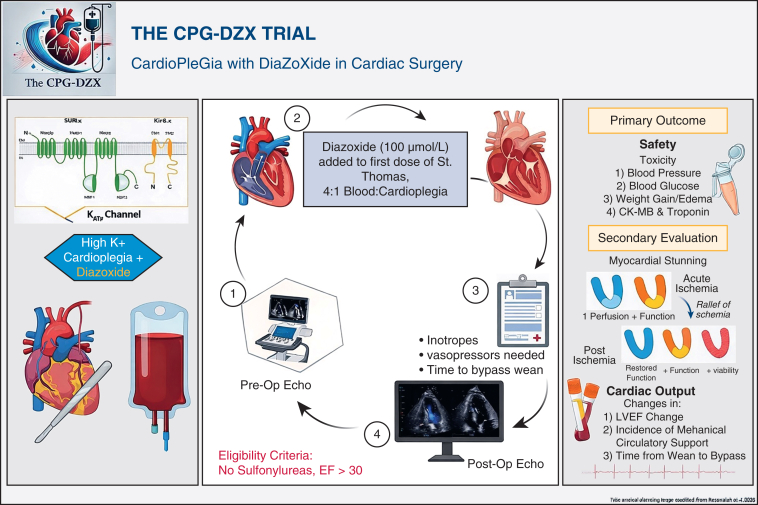
Graphical abstract showing the main components of the Cardioplegia with Diazoxide in Cardiac Surgery trial. *CK*, Creatine kinase; *EF*, ejection fraction; *K*_*ATP*_, adenosine triphosphate-sensitive potassium channel.

## Methods

### Trial Study Design and Setting

This study was a single-center safety and feasibility trial.^28^ The use of diazoxide was approved by the US FDA (IND 156146) and registered on ClinicalTrials.gov (NCT06308107). The study was approved by the Institutional Review Board of Johns Hopkins Hospital (IRB00433127, approved July 8, 2024). All patients consented to publication of their data prior to enrollment.

Diazoxide (7-chloro-3-methyl-2H-1,2,4-benzothiadiazine 1,1-dioxide; C_8_H_7_CIN_2_O_2_S; molecular weight 230.7 g/M) was previously approved by the FDA for use in its oral form (Proglycem) for primary persistent hyperinsulinemic hypoglycemia of infancy and other hypoglycemic syndromes and in its intravenous form (Hyperstat) for emergency reduction of blood pressure in severe hypertension.^29^, ^30^, ^31^

### Study Population

Thirty adult patients (age >18 years) undergoing nonemergent cardiac surgery agreed to participate. Key exclusion criteria included age <18 years, use of cardioplegia other than St Thomas, inability to consent, baseline LVEF <30%, and sulfonylurea (inhibitor of K_ATP_ channels^32^) use within the previous week. Because the definition of stunning included the duration of inotrope use, no patient on preoperative inotrope support was enrolled. Table E1 summarizes the inclusion and exclusion criteria.

### Procedure

All patients underwent cardiac surgery via standard techniques. At the time of cross-clamp placement, patients received 1 L of hypothermic, hyperkalemic St Thomas cardioplegia with 100 μmol/L of diazoxide (4:1 with patient blood) at 4°C. This was delivered via combined antegrade and retrograde routes (500 mL each) or all antegrade. Patients subsequently received St Thomas cardioplegia (4:1 with patient blood) every 20 minutes.

### Assessments

Patient age, sex, and Society of Thoracic Surgery Predicted Risk of Mortality (STS PROM) were recorded. Baseline clinical information included LVEF via transthoracic echocardiography (TTE), high-sensitivity troponin and creatine kinase (CK-MB), glucose, and weight. Intraoperatively, hemodynamic parameters, LVEF via transesophageal echocardiography (TEE), blood glucose, mean arterial pressure (MAP), time to arrest, and time to weaning from cardiopulmonary bypass (CPB) were recorded. Time to weaning from CPB was determined using the perfusion record, defined as the interval from initiation of flow reduction to cessation of CPB. Postoperatively, troponin, CK-MB, and lactate were collected every 8 hours for the first 24 hours. The postoperative need for mechanical support (intra-aortic balloon pump), inotrope and vasopressor duration, change in LVEF, and complications were recorded. Myocardial stunning was defined as need for new mechanical support and/or inotrope support for >24 hours but <72 hours (refined definition after our initial publication).^5^

Safety endpoints included systemic absorption of diazoxide or known potential side effects of oral or intravenous diazoxide administration (hypotension or hyperglycemia). No systemic drug absorption was expected, given the intracoronary route of administration. Five patients were selected at random to have peripheral venous blood samples drawn at 5 time points (preoperatively; intraoperatively after drug administration, and on postoperative days [PODs] 1, 2 and 3), and plasma was analyzed by NMS Labs (Horsham, Pa) using high-performance liquid chromatography and tandem mass spectrometry to detect diazoxide. Patients were monitored for primary safety endpoints (hyperglycemia, hypotension, and edema or weight gain) and any serious adverse events.

### Statistical Analysis

Descriptive data are presented as mean and standard deviation for continuous variables and absolute number and percentage for categorical variables. Continuous variables that were collected and compared at 2 time points included weight, MAP, glucose level, LVEF by TEE, and LVEF by TTE. These variables were analyzed using the *t* test and 2-tailed Wilcoxon matched-pairs signed-rank test. Tests were performed with a significance level of ɑ = 0.05.

## Results

### Patient Characteristics

Thirty-one patients were enrolled in 2024 and 2025. One patient did not meet the inclusion criteria after enrollment because of sulfonylurea use. The other 30 patients received diazoxide in accordance with study procedures. No patients withdrew from the study.

The 30 patients included 6 females (20%). STS PROM ranged from 0.3% to 8.0%. The most commonly performed surgery was isolated coronary artery bypass grafting (CABG; n = 15; 50%) (Table 1). The mean CPB time was 152 ± 61 minutes, and the mean aortic cross-clamp time was 108 ± 48 minutes (Table 1).

**Table 1:** Characteristics of patients who received intracoronary diazoxide added to hypothermic, hyperkalemic cardioplegia

Preoperative and surgical characteristic	Value
Female sex, n (%)	6 (20)
Age, y, mean (SD)	63.4 (12.2)
BMI, mean (SD)	28.5 (6.6)
Preoperative LVEF on TTE, mean (SD)	56.1 (8.4)
Preoperative LVEF on TEE, mean (SD)	53.3 (8.7)
STS PROM, mean (SD), range	1.4 (1.9), 0.3-8.0
Surgery performed, n (%)	
Isolated CABG	14 (46.7)
Isolated CABG, redo sternotomy	1 (3.3)
Isolated AVR	3 (10.0)
CABG, AVR, redo sternotomy	1 (3.3)
CABG, LAA clip	1 (3.3)
CABG, LAA ligation, redo sternotomy	1 (3.3)
CABG, LAA ligation, tricuspid valve repair	1 (3.3)
CABG, pulmonary valve mass excision	1 (3.3)
AVR, LAA clip	1 (3.3)
AVR, ascending aorta or root replacement	2 (6.7)
Ascending aorta replacement	2 (6.7)
Redo mitral valve replacement, aortic dissection repair, redo sternotomy	1 (3.3)
Atrial myxoma excision, LAD unroofing	1 (3.3)
CPB, min, mean (SD)	152 (61)
Aortic cross-clamp time, min, mean (SD)	108 (48)

Preoperative LVEF reported as the average based on preoperative TTE performed in the preoperative period and TEE performed intraoperatively before bypass. Time to arrest was defined as the time from first dose of cardioplegia (with diazoxide) until asystole.

*SD*, Standard deviation; *LVEF*, left ventricular ejection fraction; *TTE*, transthoracic echocardiography; *TEE*, transesophageal echocardiography; *STS PROM*, Society of Thoracic Surgeons Predicted risk of Mortality; *CABG*, coronary artery bypass grafting; *AVR*, aortic valve replacement; *LAA*, left atrial appendage; *LAD*, left anterior descending artery; *CPB*, cardiopulmonary bypass.

### Primary Outcome: Safety

There were no adverse events related to diazoxide use. One patient who was high-risk and underwent redo sternotomy with an STS PROM of 6.3% died from bowel ischemia in the postoperative period, which was determined to be unrelated to diazoxide by the study’s Data Safety and Monitoring Board.

No detectable level of diazoxide was noted in 3 patients from peripheral blood samples. Two patients had low levels of diazoxide (1400 ng/mL and 1600 ng/mL) in the first sample after surgery. No patient had a detectable level by POD 1 (Table 2). One patient underwent diazoxide serum toxicity testing owing to the need for twice the dose of induction cardioplegia (and diazoxide) to provide arrest due to intraoperative dissection and rupture of the ascending aorta at cross-clamp placement. This patient had no adverse events related to diazoxide and no detectable serum levels of diazoxide on POD 1, 2 or 3.

**Table 2:** Safety outcomes in patients who received intracoronary diazoxide as an additive to hypothermic, hyperkalemic cardioplegia

Safety outcome	Mean (SD) or n (%)	*P* value
Plasma diazoxide level measured, n (%)	6 (20)	
POD 0	Detected in 2 of 5 patients (levels: 1400 ng/mL and 1600 ng/mL)	
POD 1, 2, 3	None detected in 6 patients	
MAP, mm Hg, mean (SD)		
MAP before diazoxide	59.8 (10.7)	
MAP intraoperatively after diazoxide	62.0 (9.3)	
MAP at 48 h after diazoxide	84.9 (9.4)	
Intraoperative change in MAP, mm Hg (pre-diazoxide to immediately post-diazoxide)	2.3 (11.1)	.27
Postoperative change in MAP, mm Hg (pre-diazoxide to 48 h postoperative)	25.2 (13.3)	<.001
Glucose level, mg/dL, mean (SD)		
Glucose level at case start	113.2 (28.0)	
Glucose level after diazoxide	159.4 (29.2)	
Glucose level at 48 h after diazoxide	148.6 (32.9)	
Intraoperative change in glucose (pre-diazoxide to post-diazoxide)	46.1 (23.3)	<.001
Postoperative change in glucose (preoperative to 48 h postoperative)	36.5 (28.1)	<.001
Weight, kg, mean (SD)		
Preoperative weight, kg	87.8 (20.4)	
Postoperative change in weight, kg (preoperative to 48 h postoperative)	7.9 (2.6)	<.001

Plasma diazoxide was detectable in 2 of 5 patients who had levels measured on POD 0. Of the 6 patients who had diazoxide levels measured on POD 1, 2, and 3, diazoxide was not detected in any plasma samples. Blood pressure was monitored via arterial line (intraoperative) or noninvasive monitor (postoperative). Hyperglycemia was monitored by point-of-care or serum blood glucose measurement. For each row, the *P* value represents the paired Student *t* test comparison between the 2 time points.

*SD*, Standard deviation; *POD*, postoperative day; *MAP*, mean arterial pressure.

Hypotension is known to occur with the induction dose of hyperkalemic cardioplegia, however, cardioplegia with diazoxide was associated with a nonsignificant increase in MAP (mean change in MAP, 2.3 ± 11.1 mm Hg; *P* = .27). Glucose levels increased intraoperatively after diazoxide administration (mean change of 46 ± 23 mg/dL, *P* < .001), however, glucose remained within normal limits (mean glucose level after diazoxide 159.4 ± 29.2 mg/dL). Cardiac surgery with CPB is known to be associated with fluid shifts and weight gain.^33^ Mean weight gain was 7.9 ± 2.6 kg (*P* < .001), within the typical range of postoperative weight gain following cardiac surgery (Table 2).

No patients had a stroke or deep sternal wound infection. One patient (3%) had reoperation for bleeding, 2 patients (6.5%) had prolonged mechanical ventilation for >24 hours, and 3 patients (10%) had acute kidney injury. Two patients with acute kidney injury had complete recovery of renal function, and 1 patient required dialysis (Table 3).

**Table 3:** Postoperative morbidities in patients enrolled in the trial using STS definitions

Morbidity	n (%)	Published rates of STS morbidities, %^38^, ^39^, ^40^, ^41^
Atrial fibrillation	6 (20)	30
Stroke	0 (0)	1-6
Acute kidney injury	3 (9.7)	22-36
Prolonged ventilation (>24 h)	2 (6.5)	7-9
Deep sternal wound infection	0 (0)	1
Reoperation	1 (3.2)	3-5

STS-defined postoperative morbidities were recorded and compared to published rates.

*STS*, Society of Thoracic Surgeons.

### Additional Outcomes of Interest

The mean time to arrest was 80 ± 43 seconds, and mean time to weaning from CPB was 5.7 ± 5.3 minutes. Most patients (n = 28; 93.3%) required vasopressor or inotrope (V/I) use at the time of CPB, and 22 (73%) required V/I support at ICU arrival. By 24 hours, 25 of the 30 patients (83%) had been weaned from V/I medications. Two patients (6.7%) were on V/I for 24 to 72 hours, and these patients were defined as having myocardial stunning. Three patients (10.0%) required V/I or mechanical support for more than 72 hours (ie, 8, 9 and 12 days), and these patients were defined as requiring prolonged support. One patient (3.3%) required mechanical support (intra-aortic balloon pump) and V/I beyond 72 hours (Table 4).

**Table 4:** Outcomes in patients who received intracoronary diazoxide as an additive to hypothermic, hyperkalemic cardioplegia

Outcome	Value	*P* value
Time to arrest after cardioplegia, s	80 (43)	
Time to wean from CPB, min, mean (SD)	5.7 (5.3)	
TTE		
LVEF post-CPB, %, mean (SD)	58.2 (8.5)	
LVEF change, pre-CPB to post-CPB, %, mean (SD)	2.0 (8.6)	.195
TEE		
LVEF post-CPB, %, mean (SD)	55.4 (7.4)	
LVEF change, pre-CPB to post-CPB, %, mean (SD)	2.2 (4.9)	.024
Patients requiring support, n (%)		
V/I at CPB weaning	28 (93.3)	
V/I at ICU arrival	22 (73.3)	
V/I for 24-72 h postoperation (stunning)	2 (6.7)	
V/I for >72 h postoperation (prolonged support)	3 (10.0)	
Mechanical support	1 (3.3)	

Outcomes include 30 patients who received intracoronary diazoxide added to St Thomas cardioplegia. Change in LVEF was determined based on the difference between the patient’s preoperative and postoperative LVEF. TTE was performed preoperatively and postoperatively. TEE was performed intraoperatively, immediately before and after CPB. Time to arrest excludes 1 patient who received twice the diazoxide dose due to intraoperative aortic dissection and rupture. LVEF variables were analyzed using the paired Student *t* test.

*CPB*, Cardiopulmonary bypass; *SD*, standard deviation; *TTE*, transthoracic echocardiography; *LVEF*, left ventricular ejection fraction; *TEE*, transesophageal echocardiography; *V/I*, vasopressor or inotrope; *ICU*, intensive care unit.

The average LVEF on preoperative TTE was 56.2 ± 8.4%. The average change in LVEF on TTE (postoperative LVEF-preoperative LVEF) was 2.0 ± 8.6% (*P* = .195). The average LVEF on preoperative TEE was 58.3 ± 2.9%. The average change in LVEF on TEE (postoperative LVEF - preoperative LVEF) was 2.2 ± 4.9% (*P* = .024).

Mean peak CK-MB was 939 ± 588 U/L, and mean peak troponin was 12,531 ± 18,615 ng/L. Mean peak lactate was 5.6 ± 5.1 mmol/L, and the average time to clear lactate (defined as lactate <2.0 mmol/L) was 14.2 ± 14.6 hours (Table 5).

**Table 5:** Laboratory values for evaluating injury from global myocardial ischemia during cardiac surgery

Test	Value, mean (SD)
Creatine kinase-MB, U/L	939 (588)
Troponin, ng/L	12,530 (18,615)
Lactate, mmol/L	5.6 (5.1)
Time to lactate clearance, h	14.2 (14.6)

Mean peak laboratory values in patients who were treated with intracoronary diazoxide added to the first dose of hypothermic, hyperkalemic St Thomas cardioplegia in a safety and feasibility trial (mean ± standard deviation). Creatine kinase-MB and troponin values represent the peak values within the first 24 hours postoperatively. The lactate value indicates the peak lactate value within the first 12 hours postoperatively. Lactate clearance was defined as the first lactate level ≤2.0 mmol/L after the patient reached the intensive care unit postoperatively.

## Discussion

This study evaluated the safety and feasibility of intracoronary diazoxide in hypothermic, hyperkalemic cardioplegia in cardiac surgery patients. The primary outcome was safety and feasibility. No adverse events related to drug administration were identified. There was minimal systemic uptake of intracoronary diazoxide. Two patients had detectable levels of diazoxide after surgery, but these levels were approximately 10% of the estimated plasma concentration of diazoxide that would be observed in a patient who received a therapeutic dose of the drug.^34^

This study involved collecting additional data to confirm feasibility and to inform statistical planning for a future RCT. There is currently no accepted, clinically relevant and measurable parameter to indicate myocardial stunning. The vasoactive-inotropic score has been proposed; however, reversibility must be inherent to the definition. We developed a preliminary definition of myocardial stunning for this trial (the need for mechanical support or inotropes >24 hours^5^) and subsequently refined this definition as the need for mechanical support or inotropic support for >24 hours and <72 hours after querying a large database of patients from our institution for association with clinical outcomes and ensuring reversibility (unpublished data). Using this definition, only 6.7% of the patients in this study had stunning, a much lower prevalence than previously published.^5^ This definition will be used for Phase III trial planning.

Previous human studies using diazoxide in cardiac surgery had important differences from the present study. The first human trial of diazoxide in cardiac surgery administered diazoxide intravenously prior to cardiac surgery,^35^ and the second administered diazoxide with warm blood cardioplegia.^36^ Both of these small, randomized studies concluded that diazoxide was beneficial for cardioprotection in cardiac surgery. However, neither of these studies evaluated diazoxide added to hypothermic hyperkalemic cardioplegia. Demonstration of the safety and feasibility of diazoxide is vital, as we have demonstrated that hypothermic (and not normothermic) hyperkalemic cardioplegia is independently harmful to myocytes.^9^ In a third small study, diazoxide was administered via the aortic root to patients undergoing routine CABG. Glycocalyx proteins (hyaluronan and syndecan-1) were measured, and diazoxide was associated with improved glycocalyx integrity compared to placebo.^37^

Taken together, these previous human studies suggest potential benefit of diazoxide for cardiac surgery patients, but methodologic limitations precluded broader clinical application. Our study is the first to evaluate diazoxide added to hypothermic, hyperkalemic cardioplegia in humans.

Because it was a Phase I trial, limitations of this study include small sample size, lack of control group or randomization, and lack of blinding. These limitations were inherent to the design of this Phase 1 safety and feasibility trial. Additionally, diazoxide was administered only with hypothermic St Thomas cardioplegia, and safety and feasibility were not confirmed with other types of cardioplegia. The exclusion of patients with low LVEF (<30%) and emergent surgery limits generalizability to these patients. Finally, only 20% of the patient population was female.

Future directions will include performance of an RCT adequately powered to evaluate the efficacy of diazoxide in cardioplegia to prevent myocardial stunning during cardiac surgery. This will provide vital information regarding the use of diazoxide as a component of cardioplegia, addressing a gap in current knowledge.

## Conflict of Interest Statement

The authors reported no conflicts of interest.

The *Journal* policy requires editors and reviewers to disclose conflicts of interest and to decline handling or reviewing manuscripts for which they may have a conflict of interest. The editors and reviewers of this article have no conflicts of interest.

## Figures and Tables

**Table E1:** Inclusion and exclusion criteria for the CPG-DZX Trial

Inclusion criteria
1) Subjects ≥ 18 y of age
2) Scheduled for elective cardiac surgery with cardiopulmonary bypass and cardioplegic arrest with hypothermic hyperkalemic St. Thomas' cardioplegia
3) If female subject is of childbearing potential, she must agree to and be able to use a highly effective method of birth control (eg barrier contraceptives, hormonal contraceptives, intrauterine devices, or sexual abstinence) continuously for 6 d post-diazoxide dose
Exclusion criteria
1) Patient with diabetes mellitus on sulfonylurea medications (inhibitors of adenosine triphosphate-sensitive potassium (K_ATP_) channel)
2) Scheduled for left ventricular assist device (LVAD) or heart transplant
3) Left ventricular ejection fraction <30%
4) Pre-operative placement or planned use of mechanical circulatory support during surgery
5) Allergy to thiazide and its derivatives
6) History of gout
7) Subject is pregnant or breastfeeding
8) Subjects with seizure disorders controlled by diphenylhydantoin
9) Subjects with a history of cold agglutinins
10) Inability to provide informed consent

The CPG-DZX trial evaluated the safety and feasibility of hypothermic hyperkalemic cardioplegia with intracoronary diazoxide in cardiac surgery. Patients were enrolled in the trial based on the inclusion and exclusion criteria shown.
